# Dielectric Properties of P(VDF-TrFE-CTFE) Composites Filled with Surface-Coated TiO_2_ Nanowires by SnO_2_ Nanoparticles

**DOI:** 10.3390/polym12010085

**Published:** 2020-01-03

**Authors:** Qilong Zhang, Zhao Zhang, Nuoxin Xu, Hui Yang

**Affiliations:** State Key Lab Silicon Mat, School of Materials Science and Engineering, Zhejiang University, Hangzhou 310027, China; 21626027@zju.edu.cn (Z.Z.); xunuoxin@zju.edu.cn (N.X.); yanghui@zju.edu.cn (H.Y.)

**Keywords:** hybrid structure, composites, interfacial polarization, dielectric performances

## Abstract

Nanocomposites containing inorganic fillers embedded in polymer matrices have exhibited great potential applications in capacitors. Therefore, an effective method to improve the dielectric properties of polymer is to design novel fillers with a special microstructure. In this work, a combination of hydrothermal method and precipitation method was used to synthesize in situ SnO_2_ nanoparticles on the surface of one-dimensional TiO_2_ nanowires (TiO_2_ NWs), and the TiO_2_NWs@SnO_2_ fillers well-dispersed into the poly (vinylidene fluoride-trifluoroethylene-chlorotrifluoroethylene) [P(VDF-TrFE-CTFE)] polymer. Hybrid structure TiO_2_NWs @SnO_2_ introduce extra interfaces, which enhance the interfacial polarization and the dielectric constant. Typically, at 10 vol.% low filling volume fraction, the composite with TiO_2_NWs @SnO_2_ shows a dielectric constant of 133.4 at 100 Hz, which is almost four times that of polymer. Besides, the TiO_2_ NWs prevents the direct contact of SnO_2_ with each other in the polymer matrix, so the composites still maintain good insulation performance. All the improved performance indicates these composites can be widely useful in electronic devices.

## 1. Introduction

With the rapid growth of the microelectronics industry, electron components are integrated and miniaturized. Polymer dielectrics are widely applied in flexible displays, capacitors and energy storage devices because of good flexibility, easy processing and light-weight [1,2,3,4,5,6,7]. A relatively high dielectric constant is critical for dielectric materials. However, most polymers have low dielectric permittivity ε_r_ < 10, which hinders their application [8,9,10,11,12]. Therefore, plenty of studies have introduced ceramic particles (Pb(Zr,Ti)O_3_, BaTiO_3_, KTa_x_Nb_1−x_O_3_) as fillers into polymers to achieve a high dielectric constant [13,14,15,16,17]. Compared with spherical particles, the one-dimensional filler with a higher aspect ratio has a higher dipole moment inside, and a relatively high dielectric permittivity can be obtained at a low filling concentration [18,19]. Furthermore, theoretical calculations and experimental results show that adding a proper amount of nanowires aligned perpendicular to the external electric field direction in the composite system can help maintain or even enhance the breakdown field strength of the polymer matrix [20,21]. Among them, TiO_2_NWs have attracted more and more attention due to their moderate dielectric constant and the special role of homogenizing electric fields [22,23]. For example, Sodano et al. demonstrated that a polyvinylidene fluoride-based composite filled with 7.5 vol.% KH550 surface-modified TiO_2_ nanowires can enhance the ε_r_ of PVDF from 10 to 16 [24]. However, the ability of TiO_2_ nanowires to improve the ε_r_ is limited. When the filling volume fraction is greater than 10%, the dielectric properties of the composite material no longer improve, and may even deteriorate, showing similar characteristics to the percolation system. The reason for this result is that after reaching a certain amount of addition, the nanowires begin to overlap and aggregate with each other, and introduce defects such as holes, instead of reducing the dielectric constant and increase losses.

Tin dioxide (SnO_2_) is a semiconductor with wide band gap. Some recent studies have confirmed that nanometer-sized SnO_2_ can effectively improve the ε_r_ of polymer matrix [25,26]. Zha et al. loaded a small amount of SnO_2_ quantum dots on the surface of 100 nm BaTiO_3_, and the results showed that the ε_r_ of SnO_2_ loaded BaTiO_3_ composites did not show an advantage at the loading lower than 20 vol.%. After the volume fraction increased to 45 vol.%, BT/SnO_2_-PVDF showed a significantly improved dielectric constant (90 at 1 kHz), which is 1.4 times that of PVDF-BT [27].

In this study, a combination of hydrothermal method and precipitation method was used to synthesize in situ SnO_2_ nanoparticles on the surface of one-dimensional TiO_2_ NWs. The TiO_2_NWs @SnO_2_ fillers were successfully introduced into poly (vinylidene fluoride-trifluoroethylene-chlorotrifluoroethylene) [P(VDF-TrFE-CTFE)] with relatively high ε_r_. The small difference in dielectric constant between the matrix and the filler results in a more uniform electric field distribution, which is beneficial to maintain relatively high breakdown strength of composites. The hybrid structure TiO_2_NWs @SnO_2_ introduces additional interfaces, thereby the interfacial polarization and ε_r_ of composites is increased. Moreover, the TiO_2_ NWs prevent the direct contact of SnO_2_ from each other in polymer matrix, so the composites still maintain good insulation performance.

## 2. Materials and Methods

### 2.1. Materials

Ethanol, tin chloride dihydrate (SnCl_2_•2H_2_O), ethylene glycol (EG), hydrochloric acid, and sodium hydroxide (NaOH) were bought from Sinopharm Chemical Reagent Co., Ltd., Shanghai, China. Urea and N,N-dimethylformamide (DMF) were provided by Aladdin Industrial Corporation, Shanghai, China. Titanium dioxide (TiO_2_ < 25 nm) was supplied by Sigma-Aldrich (St. Louis, MO, USA). P(VDF-TrFE-CTFE) (64/27/9 mol.%) terpolymer was bought from Piezotech, Pierre-Benite, France.

### 2.2. Synthesis of TiO_2_@SnO_2_ Hybrid Nanopaticles

#### 2.2.1. Synthesis of TiO_2_ NWs

Firstly, 1.25 g TiO_2_ were dispersed in a mixture solution with 40 mL NaOH (10 M), 6.25 mL EG and ethanol. Secondly, the solution was transferred into a Teflon-lined autoclave and maintained at 180 °C for 48 h. The white precipitate obtained by the reaction was sufficiently washed with distilled water and immersed in a diluted 0.2 M HCl solution for 12 h. Finally, the powders were washed, dried and calcined at 700 °C for 2 h in air.

#### 2.2.2. Synthesis of TiO_2_@SnO_2_ Hybrid Nanoparticles

0.6 g TiO_2_ nanowires were distributed in 40 mL deionized water, then transferred to a three-neck round-bottom flask and heated to 60 °C. After stirring for 10 min, 0.324 mL of hydrochloric acid and stoichiometric amounts of tin chloride dihydrate and urea were added to the suspension in sequence, and kept at 60 °C for 30 min. Finally, the powders were washed, dried and calcined at 450 °C for 2 h in air. TiO_2_@SnO_2_ composite with different molar ratios of Sn:Ti (2:5; 4:5; 8:5; 16:5) were also prepared, respectively (abbreviated as TS1, TS2, TS3, and TS4).

### 2.3. Fabrication of P(VDF-TrFE-CTFE)-Based Composites

Firstly, P(VDF-TrFE-CTFE) was dissolved in DMF. Then a stoichiometric amount of TiO_2_@SnO_2_ were added with vigorously stirring and sonication. The mixture was drop-cast onto a clean substrate and dried at 80 °C overnight. Finally, the generated films followed by hot-press (2500 psi, 180 °C, 10 min). For comparison, pure polymer was also generated.

### 2.4. Characterization

The cross-section of films and the morphology of the particles were tested by scanning electron microscopy (FESEM, SU-70, Hitachi Ltd., Tokyo, Japan) and transmission electron microscopy (TEM, Tecnai G2 F20, FEI, Hillsboro, OR, USA). The crystal structure of nanoparticles and composites were performed by x-ray diffraction (XRD, EMPYREAN, PANalytical Co., Almelo, Netherlands). Escalab 250Xi x-ray photoelectron spectroscopy (XPS, Thermo Fisher Scientific, Inc., Hampton, NH, USA) was used to measure the elemental composition of nanoparticles. A Perkin–Elmer DSC-7 analyzer (Perkin–Elmer, Waltham, MA, USA) at 80–180 °C (10 °C/min) was used to measure differential scanning calorimetry (DSC). The dielectric properties were obtained by Agilent 4294A LCR Meter (Agilent, Palo Alto, CA, USA) from 10^2^–10^6^ Hz. The DC breakdown was tested at room temperature under a direct-current voltage ramp of 400 V/s (CS2674AX, Nanjing Changsheng, Nanjing, China).

## 3. Results and Discussion

### 3.1. Structure and Morphology of TiO_2_@SnO_2_ Nanoparticles

**Figure 1:**
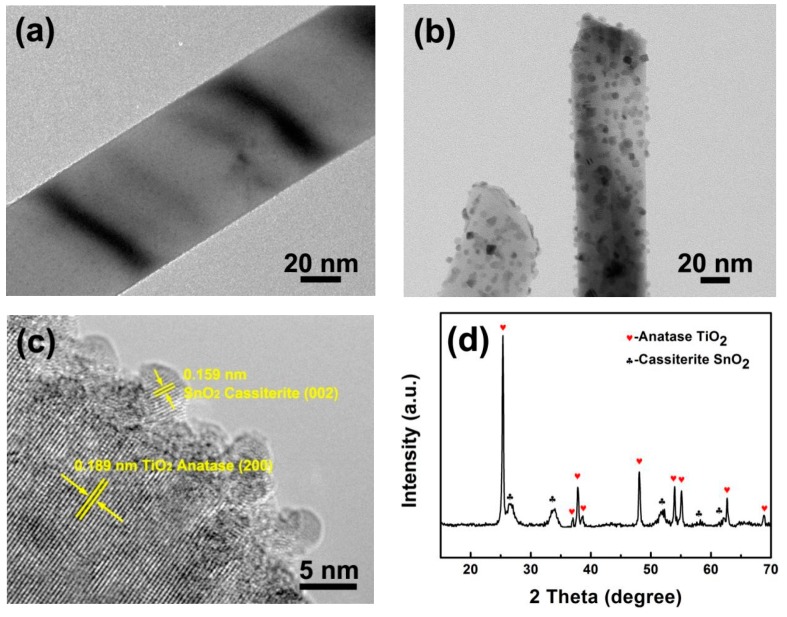
(**a**) TEM image of TiO_2_ nanowires; (**b**) TEM image, (**c**) HRTEM image and (**d**) XRD pattern of TiO_2_@SnO_2_ hybrid structure.

In Figure S1, the XRD pattern and SEM images exhibit that the as-synthesized TiO_2_ NWs possess homogenous, one-dimensional morphology without additional phases. Figure 1b shows the TEM image of TS2, which retained the original TiO_2_ nanowire morphology, but compared to the smooth pure TiO_2_ nanowires (Figure 1a), its surface was rougher and many nanoparticles were uniformly loaded. In Figure 1c, HRTEM was used to observe the nanowire/nanoparticle interface. It can be seen that the composite structure consists of two phases, where the interplanar spacing of the nanowires corresponds to the anatase phase of TiO_2_. The fringe spacing of the nanoparticles corresponds to the (002) plane of the tetragonal SnO_2_. Figure 1d is the XRD spectrum of the composite. The sharp diffraction peaks are all attributed to the anatase-type TiO_2_. In addition, diffraction peaks of other phases have been observed. The peak position is consistent with the standard spectrum (JCPDS No. 41-1445) of the tetragonal SnO_2_. It is worth mentioning that in the composite structure, all SnO_2_ nanoparticles are loaded on the surface of TiO_2_. After a long period of ultrasonic and centrifugal separation, no free particles were observed, and no exposed TiO_2_ nanowires appeared, indicating the stability of the nanoparticles and the reliability of loading method. This relatively stable structure is important for the subsequent fabrication of composite materials.

**Figure 2:**
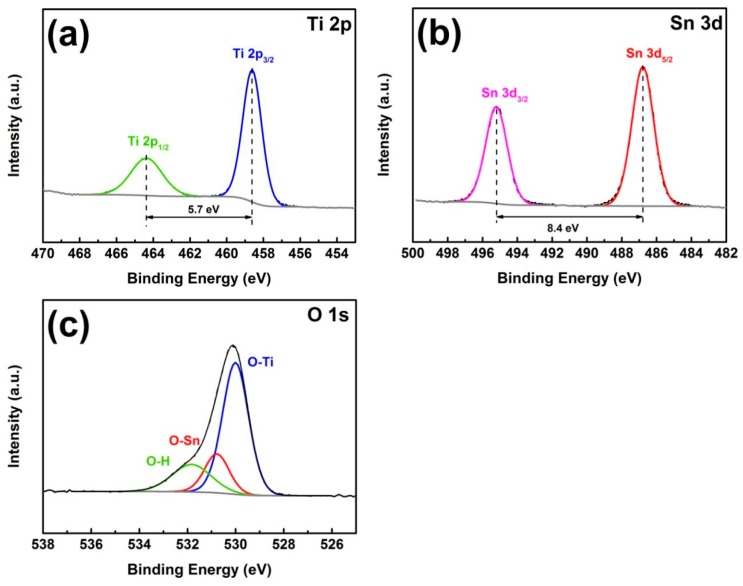
XPS spectra of TiO_2_@SnO_2_ hybrid structure. (**a**) Ti 2p, (**b**) Sn 3d, (**c**) O 1s.

The XPS test was used to further characterize the valence information of the elements in the TiO_2_@SnO_2_ composite structure. In Figure 2a, the binding energy of the Ti 2p_3/2_ and Ti 2p_1/2_ peaks are 458.6 eV and 464.3 eV, respectively. The difference in the binding energy (5.7 eV) corresponds to Ti^4+^ in TiO_2_. In the Sn 3d spectrum (Figure 2b), the peaks centered on 486.8 eV and 495.2 eV appear, corresponding to the binding energies of Sn 3d_5/2_ and Sn 3d_3/2_, respectively. At the same time, it can be seen that the shape of the peaks is more symmetrical. Figure 2c is the spectrum of O 1s. It can be observed that there is only one peak with asymmetric peak shape. After fitting it, it can be divided into three peaks. The strongest peak at 530.0 eV corresponds to O–Ti bond in TiO_2_, the second strongest peak (530.8 eV) corresponds to the O–Sn bond in SnO_2_, and the peak at 531.8 eV is connected with the hydroxyl group, which may be derived from water chemically adsorbed during sample preparation [28,29].

**Figure 3:**
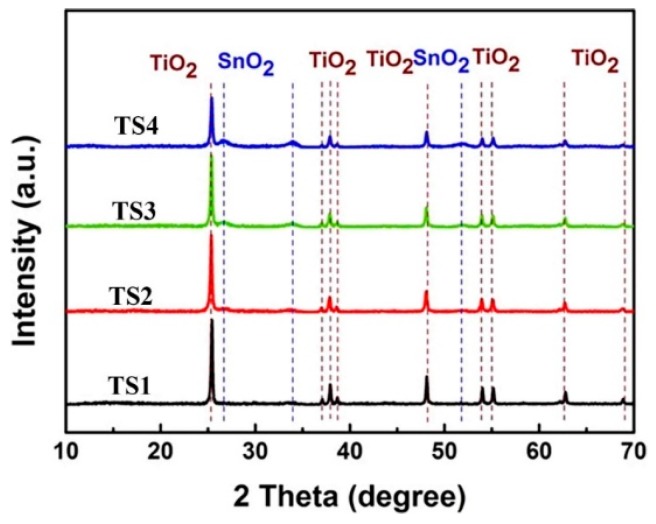
XRD patterns of TiO_2_@SnO_2_ hybrid structure.

Figure S2 illustrates the morphology of the product with various SnCl_2_/TiO_2_ molar ratios. As the initial molar ratio of Sn/Ti increased, the SnO_2_ loading on the surface of TiO_2_ nanowires also increased significantly. Moreover, SnO_2_ was well distributed on the TiO_2_ nanowires without obvious aggregations. Figure 3 shows the XRD pattern of the TiO_2_@SnO_2_ composite structure. For the TS1 sample, the main phase in the spectrum was anatase TiO_2_, and the peak of the second phase was extremely weak. With the molar ratios of Sn:Ti increased, the diffraction peak of the SnO_2_ (JCPDS No. 41-1445) gradually increased, and at the same time, the peak of the anatase TiO_2_ showed a weakening trend, which is consistent with the phenomenon of TEM. In Figure S3, it can be found that the peaks of Ti 2p and Sn 3d are separated symmetrically. The peak position and the differences between binding energy correspond to the Ti^4+^ in TiO_2_ and Sn^4+^ in SnO_2_, respectively. By increasing the Sn/Ti molar ratio, the Sn peak intensity gradually increases, while the Ti peak gradually decreases, which is consistent with XRD and TEM.

### 3.2. Morphology and Structure of TiO_2_ @SnO_2_/P(VDF-TrFE-CTFE) Composites

**Figure 4:**
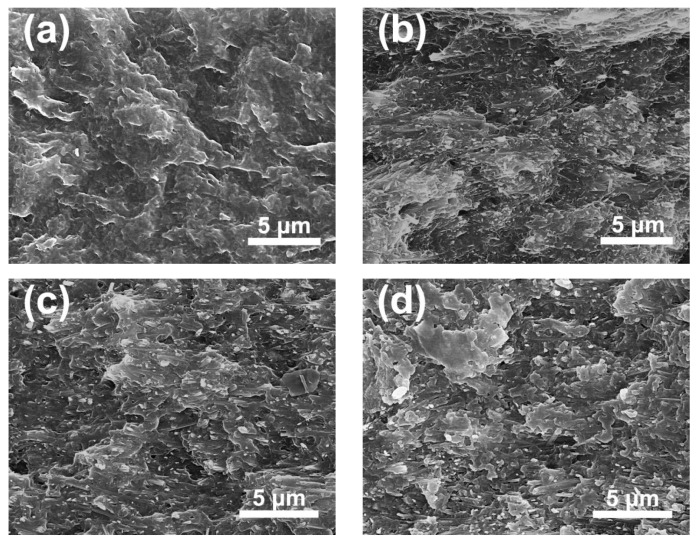

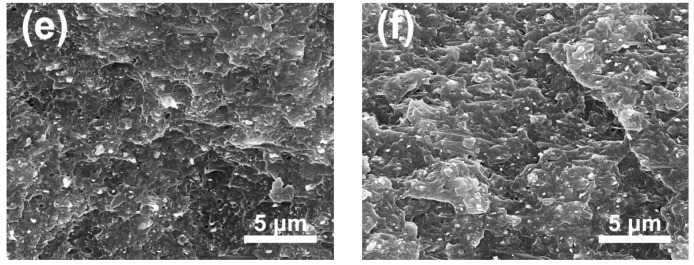
SEM images of the cross-sectional of films. (**a**) poly (vinylidene fluoride-trifluoroethylene-chlorotrifluoroethylene) [P(VDF-TrFE-CTFE)] and filled with 10 vol.% (**b**) TO, (**c**) TS2, (**d**) TS4, and 5 vol.% (**e**) TS2, (**f**) TS4.

**Figure 5:**
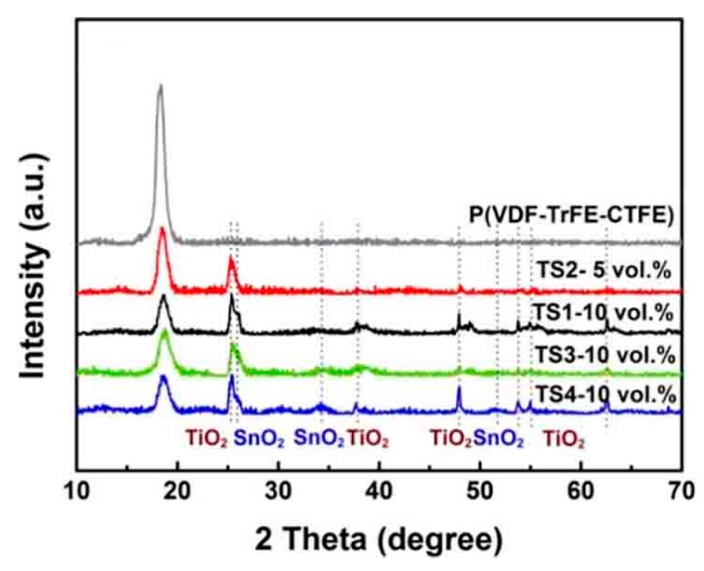
XRD patterns of polymer and its composites filled with TiO_2_@SnO_2_ hybrid structure.

Figure 4 exhibits cross-section morphology of composites. The nanocomposites exhibited dense microstructure without holes and cracks, and the interfaces between the polymer and fillers were well bonded without large-scale agglomeration. In addition, it can be observed that the arrangement direction of the filler was substantially parallel to the surface of the composites, which helps to maintain or even increase the breakdown strength (BS) of the composites [20]. Figure 5 exhibits the XRD patterns of pure polymer and nanocomposite films. In Figure 5, the peak at 18° corresponds to the compound (020) and (002) diffractions of α and γ-P(VDF-TrFE-CTFE) [30,31]. The peaks of fillers also can be observed in the composite films without secondary phase, indicating that the introduction of TiO_2_@SnO_2_ has no effect on the polymer matrix.

### 3.3. Crystallization and Melting Behavior of TiO_2_ @SnO_2_/P(VDF-TrFE-CTFE) Composites

**Figure 6:**
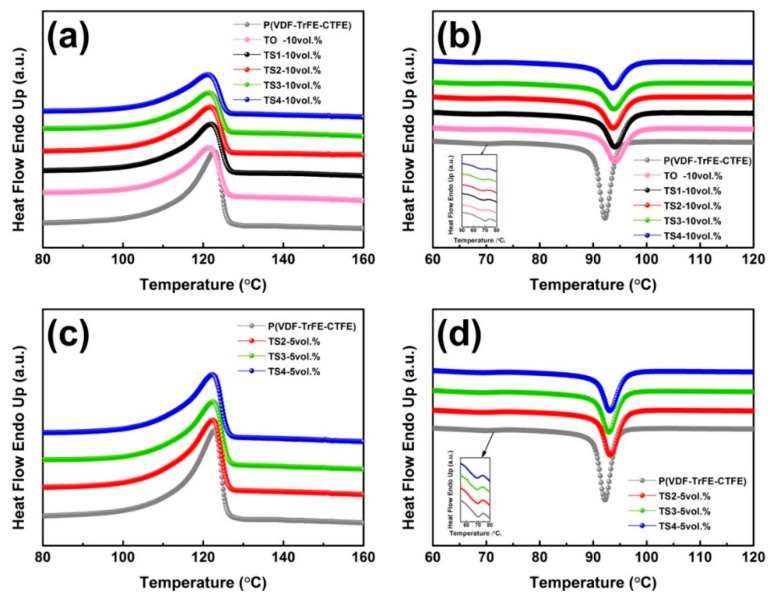
Heating curves and cooling curves of polymer and composites with (**a**,**b**) 10 vol.% and (**c**,**d**) 5 vol.% TiO_2_-based nanowires.

DSC analysis was used to explore the crystallinity (χ_c_) of the polymer, which can be calculated according to the formula:

(1) $$\chi_{c}=\frac{{\Delta H}_{m}}{\left(1-\omega \right)\times {\Delta H}_{m}^{0}}\times 100\%$$

where $\Delta H_{m}^{0}\;$ is the enthalpy of 100% crystalline P(VDF-TrFE-CTFE), $\Delta H_{m\;}$ is the heat enthalpy of the sample and ω is the mass percentage of TiO_2_@SnO_2_ nanoparticles in the polymer. Figure 6 and Table 1 show the cooling and heating curves and the crystallinities of composites. When the filling volume fraction was low, the effect of different SnO_2_ loadings on the crystallization and melting behavior of the polymer was similar. When the loading increased to 10 vol.%, the Tm of and χc generally showed a trend of rising first and then falling with the growth of SnO_2_ in the filler. Compared with the pure polymer, the crystallinity of matrix in the composite with a filling volume of 5 vol.% and 10 vol.% samples (TO, TS1) improved, and the maximum χ_c_ can be increased by 4.56% (TS1—5 vol.%). The results show that adding a suitable amount of filler in the composite system can serve as nucleating agent and promote crystallization. It can be found that in addition to the slightly obvious crystallization peak, there is a less obvious peak around 70°, indicating that the sample may undergo a very weak Curie transition around this temperature [32].

### 3.4. Dielectric Performances of TiO_2_ @SnO_2_/P(VDF-TrFE-CTFE) Composites

**Figure 7:**
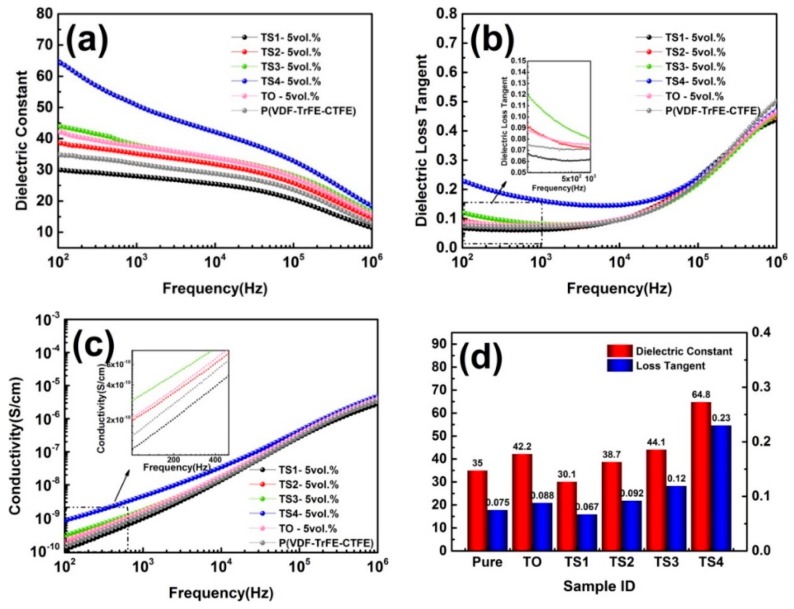
Frequency dependence of (**a**) dielectric constant, (**b**) dielectric loss, (**c**) conductivity, and (**d**) variation of dielectric constant and dielectric loss at 100 Hz of P(VDF-TrFE-CTFE) and its composites (5 vol.%).

**Figure 8:**
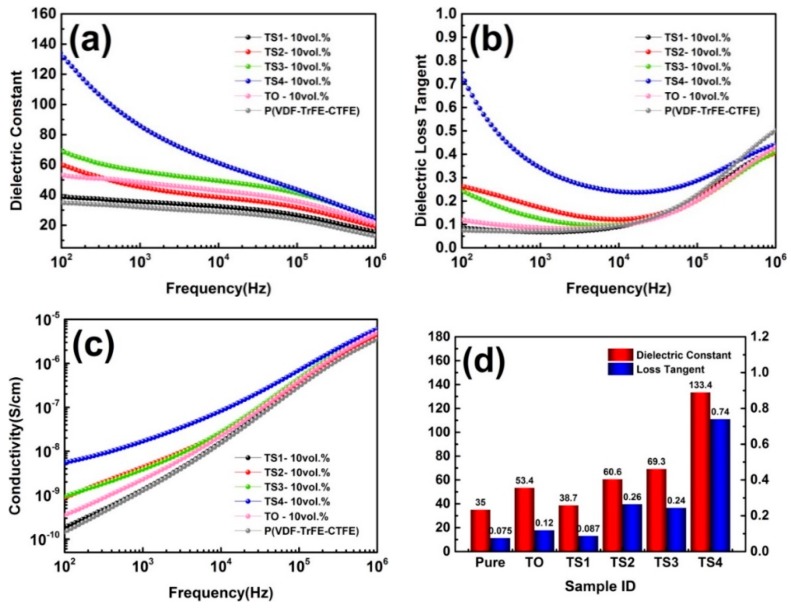
Frequency dependence of (**a**) dielectric constant, (**b**) dielectric loss, (**c**) conductivity, and (**d**) variation of dielectric constant and dielectric loss at 100 Hz of P(VDF-TrFE-CTFE) and its composites (10 vol.%).

Figure 7 displays the dielectric properties of polymer and its composites (5 vol.%). When a small amount of SnO_2_ nanoparticles were loaded on the surface of TiO_2_ nanowires (TS1), the ε_r_ of composite was lower than that of other samples, but it also showed lower dielectric loss and conductivity over the entire frequency range, even lower than the pure polymer. When the SnO_2_ was further increased, the ε_r_ showed a significantly increased. For example, the ε_r_ of the composite filled with TS4 at 100 Hz is 64.8, in contrast to 35 and 42.2 for pure polymer and the composite filled with TiO_2_. The conductivity and dielectric loss of composites also remained at a relatively low level. When the filling volume fraction of the TiO_2_ and TiO_2_/SnO_2_ increased to 10%, the dielectric performances of the sample changed. In Figure 8, when a small amount of SnO_2_ (TS1) was loaded, the ε_r_ and loss were lower than that of composite filled with TiO_2_, but greater than that of pure polymer. The filler with high SnO_2_ loading had a more significant improvement in the ε_r_ of the matrix. The composite (TS4) had the largest increase, and the ε_r_ reached 133.4 at 100 Hz. It can be seen from the above results that the effect of SnO_2_ nanoparticles introduced on the surface of TiO_2_ NWs on the dielectric properties of the composite has two sides. At low SnO_2_ loading concentration, the ε_r_ and loss of the composites were suppressed. As the load increased, the ε_r_, loss, and conductivity all gradually increased. This phenomenon is mainly related to the size and quantity of SnO_2_ [33]. On the one hand, the size of SnO_2_ particles in this work was 1–4 nm while the exciton Bohr radius of SnO_2_ particles is 2.7 nm [34]. Therefore, quantum size effect will be occurred. This effect causes the energy gap of some nanoparticles to widen, which makes charge transfer difficult, and there may be particles with reduced energy gap, which makes it easier for the charge to migrate. On the other hand, the concentration of SnO_2_ also had a significant effect on the dielectric performances of composites. At low loading concentration, the nanoparticles were far away from each other, as an isolated Coulomb Island, capturing electrons and space charges, and hindering carrier transport and inhibiting charge migration, which reduces interface polarization effects [35]. As the load increased, the distance between nanoparticles decreased. For the TS4 sample, many SnO_2_ nanoparticles loaded on the TiO_2_ nanowire formed a local network, and the distance between adjacent particles was <1 nm. At this time, the tunneling effect is very easy to occur, which causes the electrons to travel in the network [27]. Moreover, the hybrid structured TiO_2_@SnO_2_ nanoparticles introduce extra interfaces including TiO_2_@SnO_2_ interface, SnO_2_/polymer interface and TiO_2_/polymer interface. Therefore, the interface polarization and the dielectric constant are greatly improved. In addition, SnO_2_ is supported on the dispersed TiO_2_ nanowires, so SnO_2_ networks are separated from each other by a certain distance. Even if a percolation channel is formed locally, the composites still maintain good insulation performance as a whole.

**Figure 9:**
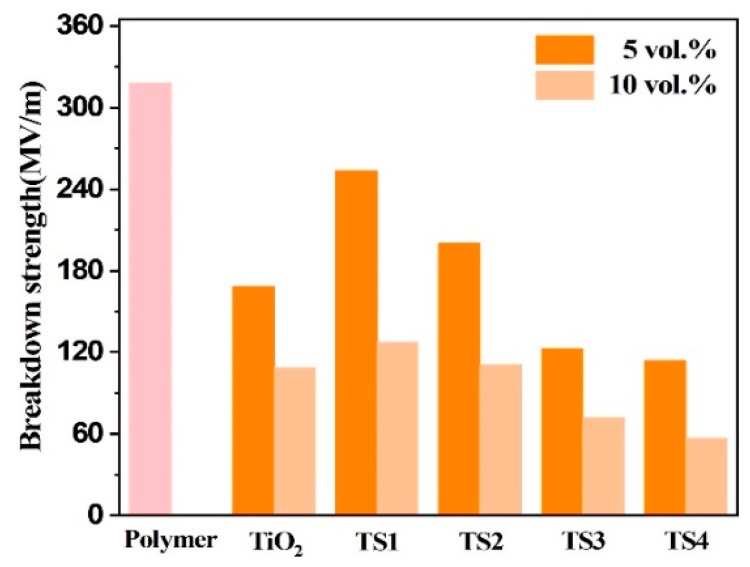
Breakdown strength of P(VDF-TrFE-CTFE) and its composites.

The breakdown strength is significant characteristic in dielectric materials, and determine the energy density of composites. The characteristic breakdown strength (CBS) of each sample could be calculated with a two parameter Weibull distribution function [36]:

(2) $$P\;=1-e^{-{\left(\frac{E}{E_{0}}\right)}^{\beta }}$$

where β is the shape parameter, P is the cumulative probability of electrical failure, *E* represents breakdown strength, and *E*_0_ is the characteristic breakdown strength (*p* = 0.632). All TiO_2_@SnO_2_/P(VDF-TrFE-CTFE) composites can withstand a high electric field exceeding 50 MV/m, as shown in Figure 9. Moreover, loading a small amount of SnO_2_ nanoparticles on TiO_2_ NWs can significantly increase the CBS of the composite. For instance, the composite filled with 5 vol.% TiO_2_ nanowires has a CBS of 168.2 MV/m, while the TS1 composite CBS with the same volume fraction is increased to higher than 250 MV/m. When the filling amount is 10 vol.%, the CBS of the TiO_2_ composite decreases to 108.5 MV/m, while the CBS of the composite filled with TS1 and TS2 is 127.3 and 110.9 MV/m, respectively. This phenomenon is consistent with the previous changes in dielectric loss, and is the result of combined effects of the quantum size and Coulomb blockade of SnO_2_ nanoparticles.

## 4. Conclusions

In conclusion, the special structure of TiO_2_ NWs/SnO_2_ fillers have successfully fabricated, and introduced into the polymer to form a novel dielectric composite. Hybrid structured TiO_2_ NWs/SnO_2_ introduce extra interfaces in the composites. The effects of TiO_2_@SnO_2_ hybrid structure with different SnO_2_ loadings on the microstructure, dielectric properties and dielectric strength of composites were explored. Typically, when a small amount of SnO_2_ nanoparticles are loaded on the surface of TiO_2_ nanowires, due to the combined effects of quantum size and Coulomb blockade, the SnO_2_ nanoparticles effectively hinder the carrier transport, thereby inhibiting the conductivity and dielectric loss of TiO_2_/P(VDF-TrFE-CTFE) composites and pure polymer, and effectively enhance the CBS of composites. As the SnO_2_ increases, the nanoparticles gradually form a local network, greatly enhancing the interface polarization effect and the ε_r_ of the composites. The composites with 10 vol.% TS4 show a dielectric constant of 133.4 at 100 Hz, which is almost four times that of the P(VDF-TrFE-CTFE). In the meantime, TiO_2_ nanowires promote the dispersion of SnO_2_ nanoparticles, so the composites maintain good insulation properties.

## Figures and Tables

**Table 1:** *T*_m_, *T*_c_ and χ_c_ of polymer and its composites.

Sample		*T*_m_ (°C)	$\chi_{c}\;(\%/{\Delta H}_{m}^{\mathrm{matrix}})$	*T*_c_ (°C)
P(VDF-TrFE-CTFE)		122.85	100	92.15
5 vol.%	TS2	122.35	104.56	93.15
	TS3	122.35	103.74	92.98
	TS4	122.35	104.14	93.15
10 vol.%	TO	121.52	100.08	94.15
	TS1	122.02	103.85	94.15
	TS2	121.85	99.29	93.82
	TS3	121.35	99.92	93.82
	TS4	121.18	96.07	93.65

## Supplementary Materials

The following are available online at https://www.mdpi.com/2073-4360/12/1/85/s1, Figure S1: SEM images of (**a,b**) H_2_Ti_3_O_7_ and (**c**) TiO_2_ nanowires; (**d**) XRD patterns of H_2_Ti_3_O_7_ and TiO_2_ nanowires. Figure S2. TEM images of the powders prepared with various SnCl_2_/TiO_2_ molar ratios: (**a**) 2:5, (**b**) 4:5, (**c**) 8:5, (**d**) 16:5. Figure S3. (**a,c,e**) TiO_2_, (**b,d,f**) Sn 3d XPS spectra of TiO_2_@SnO_2_ hybrid structure.

## References

[1] Guo M.F., Jiang J.Y., Shen Z.H., Lin Y.H., Nan C.W., Shen Y. (2019). High-energy-density ferroelectric polymer nanocomposites for capacitive energy storage: Enhanced breakdown strength and improved discharge efficiency. Mater Today.

[2] Li H., Liu F.H., Fan B.Y., Ai D., Peng Z.R., Wang Q. (2018). Nanostructured ferroelectric-polymer composites for capacitive energy storage. Small Methods.

[3] Zhou L., Jiang Y.F. (2019). Recent progress in dielectric nanocomposites. Mater. Sci. Technol..

[4] Zou K.L., Dan Y., Xu H.J., Zhang Q.F., Lu Y.M., Huang H.T., He Y.B. (2019). Recent advances in lead-free dielectric materials for energy storage. Mater. Res. Bull..

[5] Yang L.T., Kong X., Li F., Hao H., Cheng Z.X., Liu H.X., Li J.F., Zhang S.J. (2019). Perovskite lead-free dielectrics for energy storage applications. Prog. Mater. Sci..

[6] Yao Z.H., Song Z., Hao H., Yu Z.Y., Cao M.H., Zhang S.J., Lanagan M.T., Liu H.X. (2017). Homogeneous/Inhomogeneous-Structured dielectrics and their energy-storage performances. Adv. Mater..

[7] Li H., Ai D., Ren L.L., Yao B., Han Z.B., Shen Z.H., Wang J.J., Chen L.Q., Wang Q. (2019). Scalable polymer nanocomposites with record high-temperature capacitive performance enabled by rationally designed nanostructured inorganic fillers. Adv. Mater..

[8] Zhu Y.K., Jiang P.K., Zhang Z.C., Huang X.Y. (2017). Dielectric phenomena and electrical energy storage of poly (vinylidene fluoride) based high-k polymers. Chinese. Chem. Lett..

[9] Chen Q., Shen Y., Zhang S.H., Zhang Q.M. (2015). Polymer-based dielectrics with high energy storage density. Annu. Rev. Mater. Res..

[10] Huan T.D., Boggs S., Teyssedre G., Laurent C., Cakmak M., Kumar S., Ramprasad R. (2016). Advanced polymeric dielectrics for high energy density applications. Prog. Mater. Sci..

[11] Wang Y., Zhou X., Chen Q., Chu B.J., Zhang Q.M. (2010). Recent development of high energy density polymers for dielectric capacitors. IEEE Trans. Dielectr. Electr. Insul..

[12] Li H., Liu F.H., Tian H.D., Wang C., Guo Z.H., Liu P., Peng Z.R., Wang Q. (2018). Synergetic enhancement of mechanical and electrical strength in epoxy/silica nanocomposites via chemically-bonded interface. Compos. Sci. Technol..

[13] Hao Y.N., Wang X.H., Bi K., Zhang J.M., Huang Y.H., Wu L.W., Zhao P.Y., Xu K., Lei M., Li L.T. (2017). Significantly enhanced energy storage performance promoted by ultimate sized ferroelectric BaTiO_3_ fillers in nanocomposite films. Nano Energy.

[14] Tang H.X., Lin Y.R., Sodano H.A. (2012). Enhanced energy storage in nanocomposite capacitors through aligned PZT nanowires by uniaxial strain assembly. Adv. Energy. Mater..

[15] Zhang Z., Yang H., Wang H., Ding X.G., Zhang Q.L., Zhu Z.C. (2019). Enhanced dielectric properties and energy density of flexible KTa_0.2_Nb_0.8_O_3_-BaTiO_3_/P(VDF-TrFE-CTFE) nanocomposite. J. Mater. Sci. Mater. Electron..

[16] Bobić J.D., Teixeira G.F., Grigalaitis R., Gyergyek S., Petrović M.M.V., Zaghete M.A., Stojanovic B.D. (2019). PZT–NZF/CF ferrite flexible thick films: Structural, dielectric, ferroelectric, and magnetic characterization. J. Adv. Ceram..

[17] Liu S.H., Zhai J.W., Wang J.W., Xue S.X., Zhang W.Q. (2014). Enhanced energy storage density in poly(Vinylidene Fluoride) nanocomposites by a small loading of surface-hydroxylated Ba_0.6_Sr_0.4_TiO_3_ nanofibers. ACS Appl. Mater. Interfaces.

[18] Huang X.Y., Sun B., Zhu Y.K., Li S.T., Jiang P.K. (2019). High-k polymer nanocomposites with 1D filler for dielectric and energy storage applications. Prog. Mater. Sci..

[19] Liang L.Y., Kang X.L., Sang Y.H., Liu H. (2016). One-Dimensional ferroelectric nanostructures: Synthesis, properties, and applications. Adv. Sci..

[20] Tomer V., Randall C.A. (2008). High field dielectric properties of anisotropic polymer-ceramic composites. J. Appl. Phys..

[21] Hu P.H., Wang J.J., Shen Y., Guan Y.H., Lin Y.H., Nan C.W. (2013). Highly enhanced energy density induced by hetero-interface in sandwich-structured polymer nanocomposites. J. Mater. Chem. A.

[22] Yao L.M., Pan Z.B., Liu S.H., Zhai J.W., Chen H.H.D. (2016). Significantly enhanced energy density in nanocomposite capacitors combining the TiO_2_ nanorod array with poly (vinylidene fluoride). ACS Appl. Mater. Interfaces.

[23] Zhang X., Chen W.W., Wang J.J., Shen Y., Gu L., Lin Y.H., Nan C.W. (2014). Hierarchical interfaces induce high dielectric permittivity in nanocomposites containing TiO_2_@BaTiO_3_ nanofibers. Nanoscale.

[24] Tang H.X., Sodano H.A. (2013). High energy density nanocomposite capacitors using non-ferroelectric nanowires. Appl. Phys. Lett..

[25] Liu Z., Wang F.H., Zhu H. (2016). Enhanced dielectric properties of polyvinylidene fluoride with addition of SnO_2_ nanoparticles. Phys. Status Solidi R.

[26] Hoque N.A., Thakur P., Bala N., Kool A., Das S., Ray P.P. (2016). Tunable photoluminescence emissions and large dielectric constant of the electroactive poly(vinylidene fluoride-hexafluoropropylene) thin films modified with SnO_2_ nanoparticles. RSC Adv..

[27] Zha J.W., Meng X., Wang D.R., Dang Z.M., Li R.K.Y. (2014). Dielectric properties of poly(vinylidene fluoride) nanocomposites filled with surface coated BaTiO_3_ by SnO_2_ nanodots. Appl. Phys. Lett..

[28] Tian Q.H., Zhang Z.X., Yang L., Hirano S. (2014). Encapsulation of SnO_2_ nanoparticles into hollow TiO_2_ nanowires as high performance anode materials for lithium ion batteries. J. Power Sources.

[29] Bertoti I., Mohai M., Sullivan J.L., Saied S.O. (1995). Surface characterization of plasma-nitrided titanium—An xps study. Appl. Surf. Sci..

[30] Li J.J., Seok S.I., Chu B.J., Dogan F., Zhang Q.M., Wang Q. (2009). Nanocomposites of ferroelectric polymers with TiO_2_ nanoparticles exhibiting significantly enhanced electrical energy density. Adv. Mater..

[31] Lu Y.Y., Claude J., Norena-Franco L.E., Wang Q. (2008). Structural dependence of phase transition and dielectric relaxation in ferroelectric poly(vinylidene fluoride-chlorotrifluoroethylene-trifluoroethylene)s. J. Phys. Chem. B.

[32] Lu Y.Y., Claude J., Neese B., Zhang Q.M., Wang Q. (2006). A modular approach to ferroelectric polymers with chemically tunable Curie temperatures and dielectric constants. J. Am. Chem. Soc..

[33] Brus L. (1986). Electronic wave-functions in semiconductor clusters-experiment and theory. J. Phys. Chem..

[34] Lee E.J.H., Ribeiro C., Giraldi T.R., Longo E., Leite E.R., Varela J.A. (2004). Photoluminescence in quantum-confined SnO_2_ nanocrystals: Evidence of free exciton decay. Appl. Phys. Lett..

[35] Xie L.Y., Huang X.Y., Li B.W., Zhi C.Y., Tanaka T., Jiang P.K. (2013). Core-satellite Ag@BaTiO_3_ nanoassemblies for fabrication of polymer nanocomposites with high discharged energy density, high breakdown strength and low dielectric loss. Phys. Chem. Chem. Phys..

[36] Li Y., Huang X.Y., Hu Z.W., Jiang P.K., Li S.T., Tanaka T. (2011). Large dielectric constant and high thermal conductivity in poly(vinylidene fluoride)/barium titanate/silicon carbide three-phase nanocomposites. ACS Appl. Mater. Interfaces.

