# Supplementary material for: Dielectric Properties of P(VDF-TrFE-CTFE) Composites Filled with Surface-Coated TiO2 Nanowires by SnO2 Nanoparticles
Source: Polymers (Basel). 2020 Jan 3;12(1):85. doi: 10.3390/polym12010085 (PMC7023657; doi:10.3390/polym12010085)
Supplement: Supplementary file 1 [file polymers-12-00085-s001.pdf]

Electronic Supplementary Information for:

Dielectric Properties of P(VDF-TrFE-CTFE) Nanocomposites Filled with Surface-coated TiO_2 Nanowires by SnO_2 Nanoparticles

Qilong Zhang*, Zhao Zhang, Nuoxin Xu, Hui Yang

[1] School of Materials Science and Engineering, State Key Lab Silicon Mat, Zhejiang University, Hangzhou 310027, P. R. China

* Correspondence: mse237@zju.edu.cn

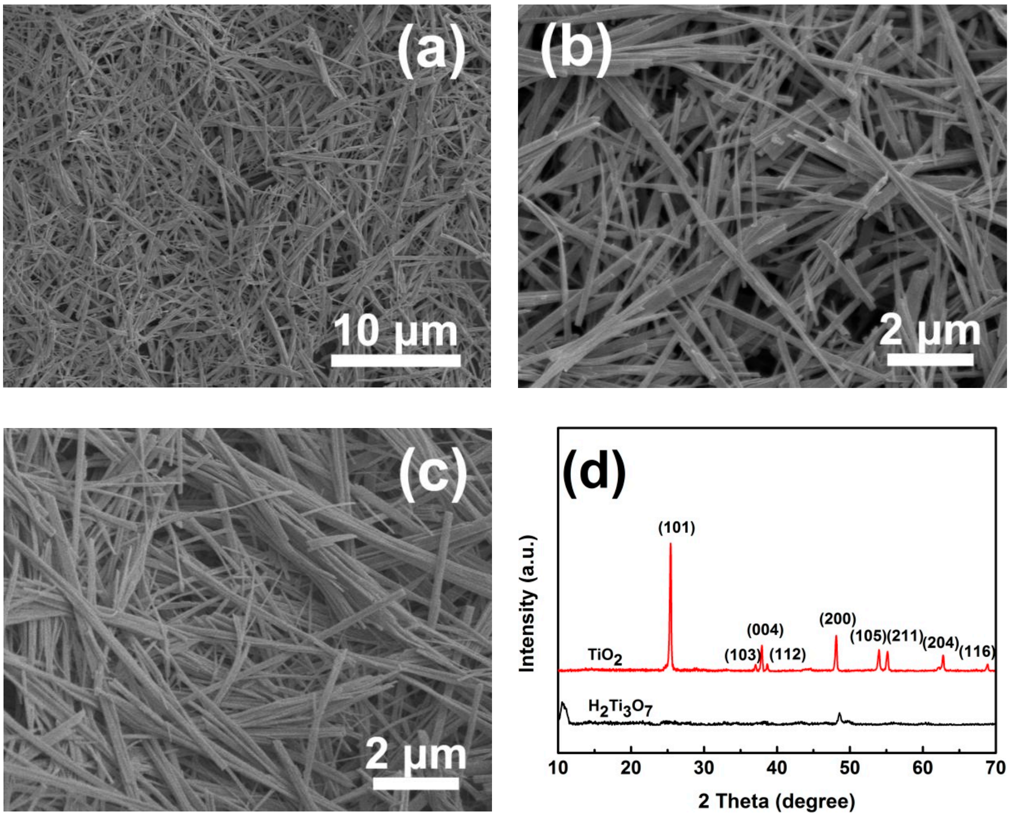

Figure S1. SEM images of (a)(b) $H_2Ti_3O_7$ and (c) TiO_2 nanowires; (d)XRD patterns of $H_2Ti_3O_7$ and TiO_2 nanowires.

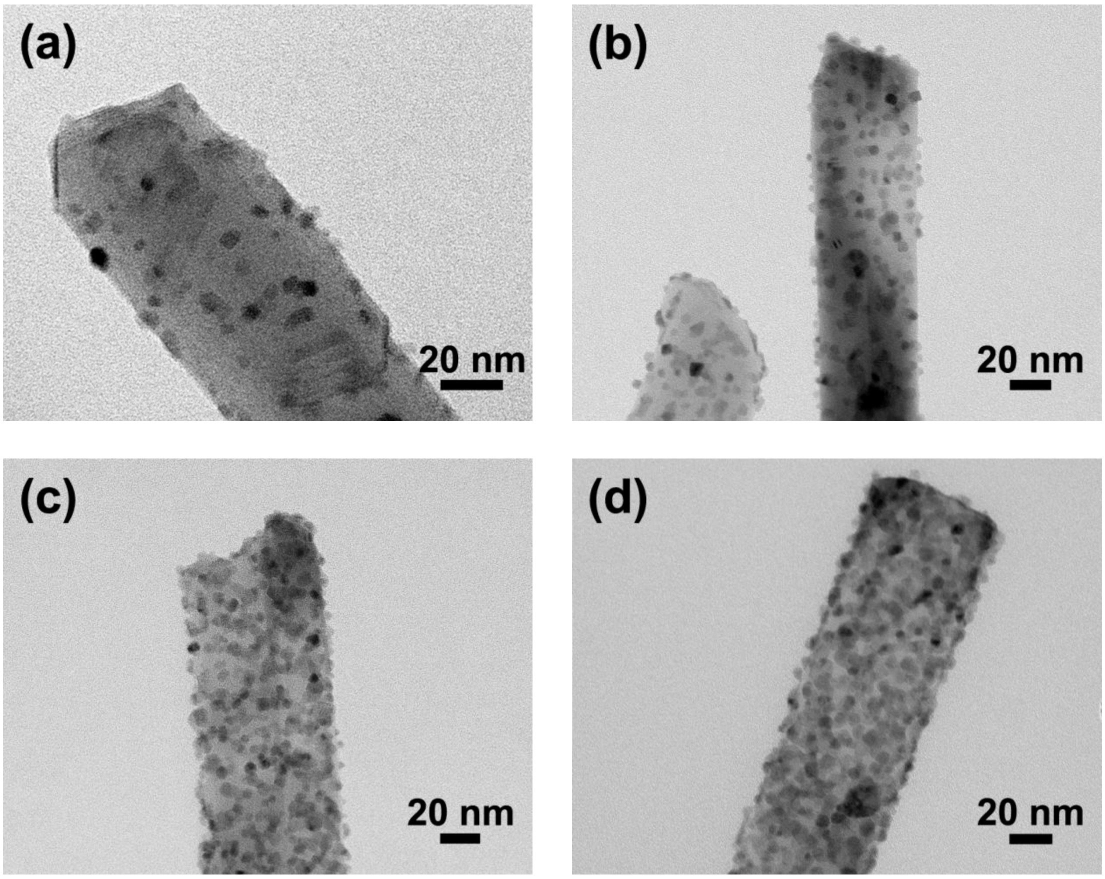

Figure S2. TEM images of the powders prepared with various $SnCl_2/TiO_2$ molar ratios: (a) 2:5, (b) 4:5, (c) 8:5, (d) 16:5.

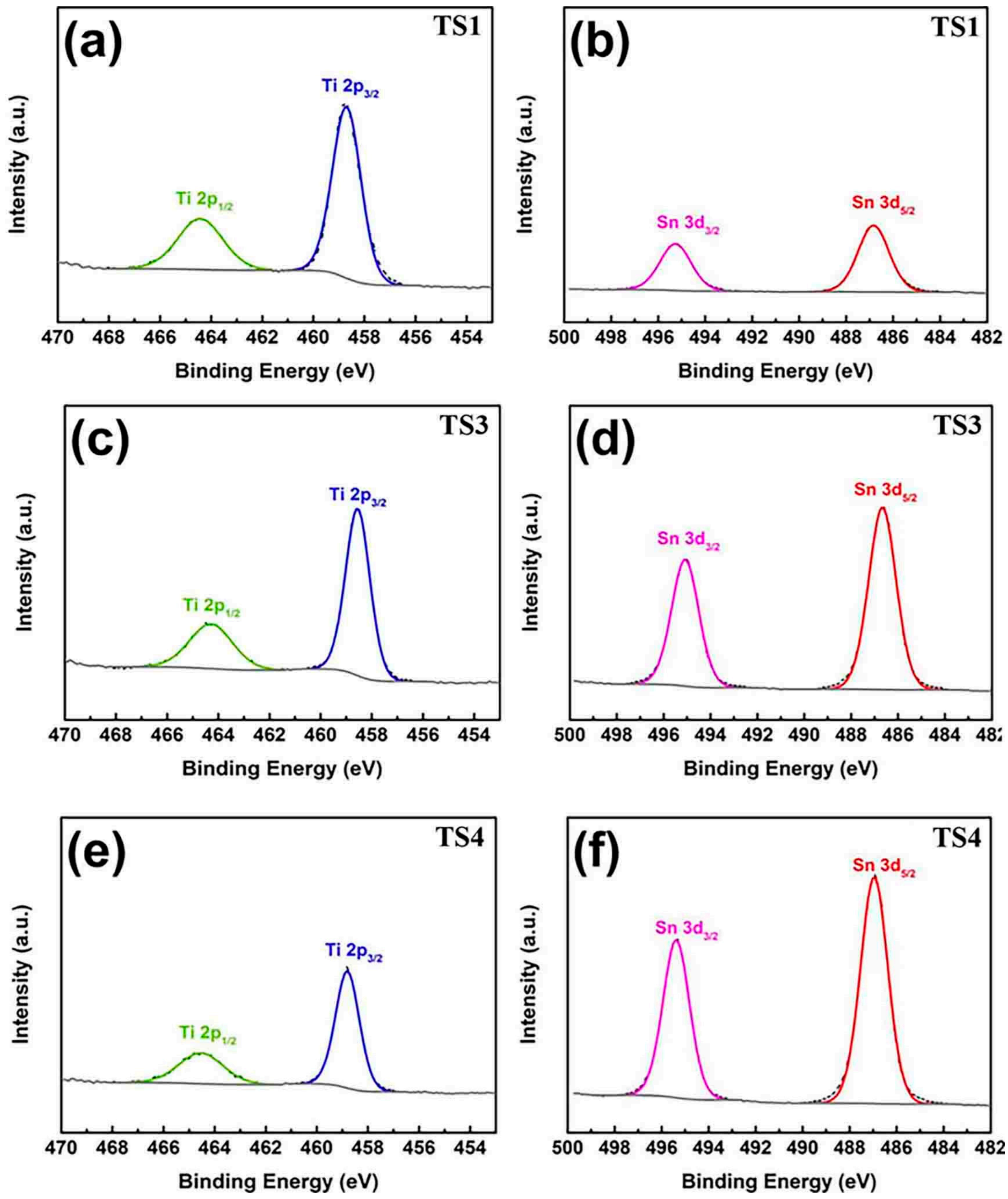

Figure S3. (a)(c)(e)Ti 2p, (b)(d)(f)Sn 3d XPS spectra of TiO_2@SnO_2 hybrid structure.
